# Pulmonary *Nocardia paucivorans* Infection Mimicking Lung Cancer in an Immunocompetent Patient: A Case Report

**DOI:** 10.1155/crdi/4621773

**Published:** 2026-07-02

**Authors:** Mario Mekhail, Kevin Shiah, Arunmozhi Aravagiri, Bruce Weng, Made Sutjita

**Affiliations:** ^1^ Division of Pulmonary and Critical Care Medicine, Department of Internal Medicine, Loma Linda University, Loma Linda, California, USA, llu.edu; ^2^ Department of Internal Medicine, Division of Infectious Diseases, Riverside University Health System Medical Center, Moreno Valley, California, USA, ruhealth.org

**Keywords:** lung cancer mimic, MALDI-TOF MS, *Nocardia paucivorans*, pulmonary nocardiosis, trimethoprim–sulfamethoxazole

## Abstract

**Background:**

Pulmonary nocardiosis is uncommon and usually affects immunocompromised hosts, but it can occur in immunocompetent patients and mimic primary lung malignancy clinically and radiographically.

**Case Presentation:**

A 52‐year‐old immunocompetent woman presented with one month of cough, hemoptysis, and mild shortness of breath after repeated hiking exposures in Mexico and Central California. Chest CT showed bilateral apical ground‐glass opacities and a spiculated, pleural‐based mass involving the right upper and lower lobes, raising concern for primary lung cancer. HIV testing was negative; HbA1c was 5.1%; and initial sputum, blood, and acid‐fast bacilli studies were negative. CT‐guided biopsy showed acute and chronic inflammation, necrosis, and stromal fibrosis without well‐formed granulomas. Gram stain identified Gram‐positive rods. Tissue culture grew *Nocardia paucivorans*, identified by MALDI‐TOF MS and confirmed by DNA sequencing. Susceptibility testing showed TMP‐SMX susceptibility. She received prolonged oral TMP‐SMX from November 2022 through December 2024, with resolution of cough and hemoptysis. Serum creatinine rose mildly during therapy but normalized without interruption. Follow‐up CT showed improvement in the dominant mass, with residual bilateral upper‐lobe abnormalities favored to represent postinflammatory fibrosis.

**Conclusion:**

Pulmonary *Nocardia paucivorans* infection can closely mimic lung cancer, even in immunocompetent hosts. When noninvasive testing is unrevealing, tissue biopsy may be necessary for diagnosis. Radiological follow‐up after treatment is warranted to exclude occult malignancy masked by infection.

## 1. Introduction

*Nocardia* species are aerobic, branching, filamentous, Gram‐positive bacteria found in soil, water, and decaying organic matter. Human infection is usually acquired through inhalation or direct inoculation. Pulmonary disease is the most common manifestation, accounting for approximately 75% of nocardiosis cases, and is classically associated with impaired cell‐mediated immunity, chronic corticosteroid use, malignancy, organ transplantation, and advanced HIV infection. However, pulmonary nocardiosis can also occur in immunocompetent hosts and may be difficult to recognize because its clinical and radiographic findings overlap with tuberculosis, fungal infection, and primary lung cancer [1, 2]. In a large comparative study, 40% of patients with culture‐proven nocardiosis were nonimmunocompromised, and 1‐year all‐cause mortality was significantly lower in this group (7% vs. 27%; *p* = 0.01) [3].

Clinical presentation is typically subacute, with cough, fever, and dyspnea; hemoptysis is less common. Extrapulmonary dissemination, most notably to the central nervous system (CNS), occurs in 20%–40% of cases and is more frequent in immunocompromised hosts [4, 5]. The differential diagnosis includes primary lung malignancy, pulmonary tuberculosis, fungal infections such as aspergillosis and histoplasmosis, and pulmonary actinomycosis. Diagnosis relies on culture of respiratory specimens, including sputum, bronchoalveolar lavage (BAL) fluid, or tissue biopsy, with Gram stain, modified acid‐fast stain, and susceptibility testing. Species identification can be achieved by MALDI‐TOF MS, ideally with molecular confirmation for clinically significant or uncommon isolates [6, 7]. The reference standard for *Nocardia* species identification remains molecular sequencing, and the preferred method for antimicrobial susceptibility testing is broth microdilution [8]. Treatment generally centers on TMP‐SMX for susceptible isolates, with duration tailored to disease extent and host immune status [8–10].

*Nocardia paucivorans* is an uncommon species first described in 2000 and has been reported in pulmonary, cutaneous, and CNS infections [11, 12]. Species‐level identification is clinically relevant because antimicrobial susceptibility patterns differ across *Nocardia* species, including variable sulfonamide resistance patterns [8, 9, 13]. We describe pulmonary *Nocardia paucivorans* infection in an immunocompetent woman who presented with a spiculated pulmonary mass highly concerning for malignancy.

## 2. Case Presentation

A 52‐year‐old woman presented with a 1‐month history of cough, hemoptysis, and mild shortness of breath. She reported frequent hiking exposures in Mexico and Central California. Physical examination was notable only for bilateral scattered apical rales and crackles; the remainder was unremarkable. She had no history of diabetes mellitus, HIV infection, malignancy, organ transplantation, or chronic immunosuppressive therapy. HIV testing was negative, and HbA1c was 5.1%.

Initial chest CT demonstrated bilateral apical ground‐glass and consolidative opacities (Figure 1A), as well as a spiculated, pleural‐based mass in the posterior right lung straddling the major fissure and involving the right upper and lower lobes (Figure 1B). Imaging raised concern for primary lung malignancy, although infectious etiologies, including mycobacterial and fungal disease, remained in the differential diagnosis.

**FIGURE 1:**
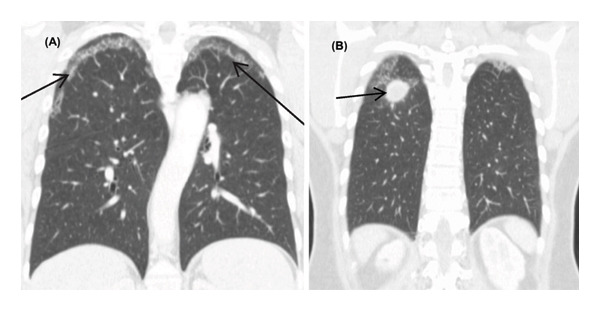
Initial chest CT findings at presentation. (A) Bilateral apical ground‐glass opacities and consolidation, raising concern for granulomatous infection, including tuberculosis (arrows). (B) Well‐defined mass in the right upper lobe (arrow), which demonstrated spiculated margins and pleural‐based morphology on axial imaging, highly concerning for primary lung malignancy.

Initial noninvasive testing was unrevealing. Sputum cultures, blood cultures, and acid‐fast bacilli studies were negative. Bronchoscopy with BAL was not performed before biopsy; given the peripheral, pleural‐based location of the mass and persistent concern for malignancy, CT‐guided percutaneous biopsy was pursued as the most direct diagnostic approach. In cases where a lesion is centrally located or endobronchially accessible, bronchoscopy with BAL should be considered an initial diagnostic step.

Histopathologic examination showed focal acute and chronic inflammation, necrosis, and stromal fibrosis without well‐formed granulomas. Gram stain demonstrated Gram‐positive branching rods. No fungal organisms were identified on special stains. Tissue culture from the biopsy grew *Nocardia paucivorans*. Initial species identification was performed by MALDI‐TOF MS. Although MALDI‐TOF MS has demonstrated high accuracy for common *Nocardia* species, its performance is limited for rare or low‐prevalence species such as *Nocardia paucivorans* because of incomplete reference library coverage; in one study, concordance between MALDI‐TOF MS and molecular methods was only 11% for low‐prevalence *Nocardia* species [6, 7]. Confirmatory 16S rRNA or secA1 gene sequencing may therefore be essential for definitive species‐level identification in clinically significant or uncommon isolates. The isolate was confirmed by DNA sequencing at a reference laboratory. Antimicrobial susceptibility testing was performed using broth microdilution at the reference laboratory (Table 1).

**TABLE 1:** Antimicrobial susceptibility profile of the *Nocardia paucivorans* isolate.

Antimicrobial agent	MIC (μg/mL)	Interpretation
Amikacin	≤ 0.5	Susceptible
Ceftriaxone	2	Susceptible
Ciprofloxacin	0.25	Susceptible
Tobramycin	≤ 2	Susceptible
Trimethoprim–sulfamethoxazole	0.12	Susceptible
Ampicillin	32	Resistant
Imipenem	8	Intermediate

The isolate demonstrated susceptibility to TMP‐SMX, amikacin, ciprofloxacin, ceftriaxone, and tobramycin, with intermediate susceptibility to imipenem and resistance to ampicillin (Table 1). TMP‐SMX was selected as the first‐line therapy given its established efficacy against susceptible *Nocardia* isolates, excellent oral bioavailability, and favorable tolerability profile for outpatient management [8, 9].

The patient started oral TMP‐SMX in November 2022. Initial weight‐based dosing of the trimethoprim component was approximately 8 mg/kg/day, corresponding to five double‐strength tablets daily. She was later transitioned to two double‐strength tablets twice daily. A subsequent dose reduction to one double‐strength tablet twice daily was attempted; however, the patient still had a cough and shortness of breath. Follow‐up CT on March 15, 2024, showed persistent bilateral upper‐lobe consolidations, and the dose was increased again to two double‐strength tablets twice daily due to concern for an incomplete radiographic response.

Published recommendations for pulmonary nocardiosis without CNS involvement generally support at least 6–12 months of therapy in immunocompetent patients, but treatment duration should be individualized according to clinical and radiographic response [8, 9, 14]. CNS involvement was considered unlikely on clinical grounds, as the patient had no neurological symptoms throughout the illness. Although routine brain imaging is strongly recommended in immunocompromised patients with nocardiosis, given that up to one‐third of patients with CNS involvement may lack neurological symptoms, the role of routine neuroimaging in immunocompetent patients with isolated pulmonary disease is less well established [8]. In cases where CNS disease cannot be excluded clinically, dedicated neuroimaging is warranted, as CNS involvement mandates at least 12 months of therapy and often requires combination regimens [4, 5].

Her cough and hemoptysis resolved with treatment. She continued therapy through December 9, 2024. Follow‐up CT showed persistent bilateral upper‐lobe ground‐glass and consolidative changes, which were favored to represent postinflammatory fibrosis rather than ongoing active infection (Figure 2A) with interval improvement in the pleural‐based mass (Figure 2B). The clinical timeline from diagnosis to completion of therapy is summarized in Table 2.

**FIGURE 2:**
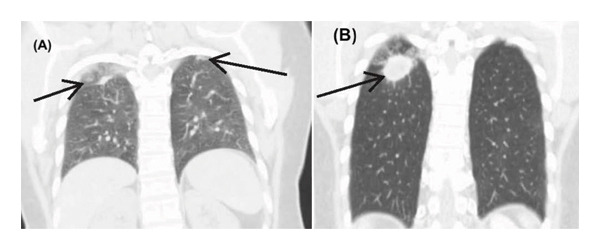
Follow‐up chest CT after completion of TMP‐SMX therapy. (A) Interval improvement in the dominant right‐sided mass (arrows). (B) Residual bilateral upper‐lobe abnormalities favored to represent postinflammatory fibrosis (arrows).

**TABLE 2:** Clinical timeline from diagnosis to follow‐up.

Date/period	Clinical event
November 2022	Diagnosis established from CT‐guided lung biopsy tissue culture; TMP‐SMX initiated at ∼8 mg/kg/day trimethoprim component (five double‐strength tablets daily)
Early 2023	Dose transitioned to two double‐strength tablets twice daily
2023‐2024	Dose‐reduction trial to one double‐strength tablet twice daily; persistent cough and shortness of breath
March 15, 2024	CT showed persistent bilateral upper‐lobe consolidations; dose re‐escalated to two double‐strength tablets twice daily
December 9, 2024	Antimicrobial therapy completed
Follow‐up	Symptoms resolved; residual imaging abnormalities favored to represent postinflammatory fibrosis; surveillance imaging ongoing

Treatment was generally well tolerated. Renal function was monitored with periodic serum creatinine measurements throughout therapy. Serum creatinine rose mildly from 0.9 mg/dL (79.6 µmol/L) to 1.21 mg/dL (107.0 µmol/L) during treatment. This rise is consistent with trimethoprim’s well‐characterized inhibition of renal tubular creatinine secretion via Organic cation transporter 2 (OCT2), thereby reducing tubular creatinine excretion and raising serum creatinine without a true decline in glomerular filtration rate [15]. No dose modification was required for renal function, and creatinine normalized during the course of therapy.

## 3. Discussion

Pulmonary nocardiosis remains an important diagnostic consideration in patients with subacute respiratory symptoms and abnormal thoracic imaging, particularly when routine studies are negative. Although nocardiosis is more commonly seen in immunocompromised patients, it can also occur in immunocompetent individuals [1–3]. Given the patient’s history of frequent hiking, exposure to environmental soil likely contributed to disease acquisition.

Radiographic findings in pulmonary nocardiosis are variable and may include nodules, masses, cavitary lesions, ground‐glass opacities, or consolidation [1, 10]. In our patient, the dominant lesion was a spiculated pleural‐based mass, an appearance strongly suggestive of lung cancer. The bilateral apical opacities further broadened the differential to include tuberculosis and other granulomatous infections. Prior reports have highlighted the clinical and radiographic overlap between pulmonary nocardiosis and lung malignancy, reinforcing the importance of tissue diagnosis in ambiguous cases [16, 17].

Pulmonary actinomycosis also warrants consideration because it can mimic lung malignancy with mass‐like consolidation, pleural involvement, and constitutional symptoms [16, 17]. Like pulmonary nocardiosis, it is caused by a filamentous organism and requires prolonged antimicrobial therapy, although the regimens differ: penicillin‐based regimens are preferred for actinomycosis, whereas TMP‐SMX is commonly used for nocardiosis [17]. Both diagnoses should be considered when a lung biopsy reveals chronic suppurative inflammation without an immediately identifiable etiology.

In this patient, the initial sputum, blood, and acid‐fast studies were negative, and the diagnosis was established only after CT‐guided biopsy. Species‐level identification is clinically useful because antimicrobial susceptibility patterns differ across *Nocardia* species [8, 9, 13]. Here, identification by MALDI‐TOF MS and confirmatory DNA sequencing provided robust microbiologic support for the diagnosis.

Published reports of *Nocardia paucivorans* remain limited, but available data suggest that it can cause pulmonary and disseminated disease, including CNS involvement [11, 12, 18]. Gray et al. described 33 patients with *Nocardia paucivorans* isolates over a 20‐year period and found that at least 30% had disseminated disease, indicating that this species can spread beyond the lung [11]. Hammoud et al. reported pulmonary, iliopsoas, and CNS involvement in an immunocompetent host, with 16S rRNA gene sequencing expediting diagnosis several weeks before culture positivity [18]. Samannodi reported disseminated *Nocardia paucivorans* infection in an immunocompetent patient and noted that a 6–12‐month course of TMP‐SMX is generally appropriate for susceptible isolates without CNS involvement [19]. A comparative summary of published cases is provided in Table 3.

**TABLE 3:** Summary of selected published cases of *Nocardia paucivorans* infection.

Reference	Year	Age/sex	Immune status	Presentation	Imaging	Diagnosis	Treatment	Outcome
Eisenblätter et al. [12]	2002	NR	Chronic lung disease	Pulmonary source; CNS/meningeal spread	Brain MRI: ring‐enhancing lesions	CSF culture; susceptibility testing	TMP‐SMX + additional agents	Cerebral relapse
Wellinghausen et al. [20]	2002	Multiple	Mixed	Pulmonary/other manifestations	NR	16S rRNA sequencing	TMP‐SMX‐based	Variable
Gray et al. [11]	2007	53M; 33 isolates	Immunocompetent	Pulmonary infection with CNS/cutaneous spread	NR	Culture + molecular ID	TMP‐SMX‐based	≥ 30% disseminated
Wegerle et al. [21]	2007	NR	Immunocompetent	Pulmonary nocardiosis	NR	Culture; TMP‐SMX resistance	Alternative agents	Improved
Hammoud et al. [18]	2014	50M	Immunocompetent	Cough, confusion, weight loss; pulmonary + CNS disease	Pulmonary/iliopsoas masses; brain lesions	16S rRNA sequencing	12 months antibiotics	Lesions resolved
George et al. [22]	2019	66M	Cancer, CLL	Empyema; right apical cavitary mass	Cavitary mass; bilateral infiltrates	MALDI‐TOF MS	TMP‐SMX + imipenem; drainage	Improved
Samannodi [19]	2021	NR	Immunocompetent	Disseminated nocardiosis	NR	Culture	TMP‐SMX 6–12 months	Resolved
Wintheiser et al. [23]	2021	44M	Immunocompetent	Fever, headache, weight loss; lung + CNS disease	FDG‐avid lung mass; brain abscesses	Broad‐range 16S PCR	IV TMP‐SMX + amikacin + imipenem	NR
Nour et al. [24]	2024	NR (elderly male)	Gold miner	LRTI mimicking tuberculosis	Pulmonary infiltrates	16S rRNA sequencing	NR	Fatal
Current report	—	52F	Immunocompetent	Cough, hemoptysis, mild dyspnea; lung cancer mimic	Bilateral apical GGO/consolidation + pleural‐based mass	MALDI‐TOF MS + DNA sequencing	TMP‐SMX, Nov 2022–Dec 2024	Clinical resolution; residual fibrosis

*Note:* CSF, cerebrospinal fluid; FDG, fluorodeoxyglucose; IV, intravenous; TMP‐SMX, trimethoprim–sulfamethoxazole.

Abbreviations: CLL, chronic lymphocytic leukemia; CNS, central nervous system; CT, computed tomography; GGO, ground‐glass opacity; MALDI‐TOF MS, matrix‐assisted laser desorption/ionization time‐of‐flight mass spectrometry; MRI, magnetic resonance imaging; NR, not reported; PCR, polymerase chain reaction; RLL, right lower lobe; RUL, right upper lobe.

TMP‐SMX remains a mainstay of therapy for susceptible isolates, although treatment duration is often prolonged and should be individualized based on disease burden, response, and host factors [8, 9, 14]. In our patient, therapy was extended beyond the usual 6–12 months range because of residual symptoms, including cough and shortness of breath, and serial CT imaging continued to show persistent bilateral upper‐lobe consolidations, including after an attempted TMP‐SMX dose reduction, raising concern for incomplete radiographic response. The patient nonetheless improved clinically with prolonged therapy, and imaging demonstrated a reduction of the dominant lesion; residual upper‐lobe abnormalities were ultimately favored to represent postinflammatory fibrosis. Emerging data from Attias et al. suggest that shorter treatment durations (≤ 90 days) may be safe in selected patients with solitary pulmonary nocardiosis and good clinical response, although this remains based on retrospective data and requires prospective validation [25].

Regarding TMP‐SMX dosing intensity, a recent multicenter retrospective cohort study by Yetmar et al. (*n* = 91) compared outcomes across high‐dose (> 10 mg/kg/day trimethoprim component), intermediate‐dose (5–10 mg/kg/day), and low‐dose (< 5 mg/kg/day) TMP‐SMX regimens for nondisseminated pulmonary nocardiosis [26]. Low‐ and intermediate‐dose TMP‐SMX were not associated with worse outcomes compared to high‐dose therapy; recurrence risk was similar across all groups. Notably, 66.7% of patients in the high‐dose group required dose adjustment or early discontinuation compared to approximately 25% in the intermediate‐ and low‐dose groups (*p* = 0.001) [26]. These findings suggest that historically used high‐dose TMP‐SMX may not be necessary for isolated pulmonary nocardiosis, although the data are retrospective and do not apply to disseminated or CNS disease. In our patient, initial dosing at approximately 8 mg/kg/day fell within the intermediate‐dose range, and the subsequent maintenance dose of two double‐strength tablets twice daily was consistent with this emerging evidence.

Pulmonary nocardiosis not only can radiographically resemble lung malignancy but can also obscure an underlying occult malignancy [17]. Radiological follow‐up after treatment completion is therefore mandatory, particularly when residual imaging abnormalities persist. In our patient, residual bilateral upper‐lobe changes were favored to represent postinflammatory fibrosis; however, continued surveillance imaging is planned to confirm stability and definitively exclude an underlying malignancy.

To our knowledge, this is one of the few published cases of pulmonary *Nocardia paucivorans* infection presenting as a spiculated mass that closely mimics primary lung cancer in an immunocompetent host, underscoring the diagnostic challenge this species can pose.

This case has several limitations, including the unavailability of representative histopathology or microbiology images for publication and the need for continued radiological surveillance to confirm the stability of residual abnormalities.

## 4. Conclusion

Pulmonary *Nocardia paucivorans* infection can present as a spiculated lung mass and closely mimic primary lung cancer, even in immunocompetent patients. When initial microbiologic studies are negative and imaging remains concerning, tissue biopsy may be essential for diagnosis. Early recognition and species‐level identification, particularly with molecular confirmation, facilitate targeted therapy and help avoid treatment delays. This case reinforces the importance of including nocardiosis in the differential diagnosis of a dominant lung mass in patients without overt immunosuppression and highlights the need for radiological follow‐up after treatment to exclude occult malignancy.

## Author Contributions

Mario Mekhail contributed to the literature review, manuscript drafting, and revision. Kevin Shiah contributed to case identification, data collection, and manuscript preparation. Arunmozhi Aravagiri contributed to data acquisition and manuscript review. Bruce Weng contributed to the interpretation of microbiologic data and manuscript review. Made Sutjita supervised the case, contributed to study conception, and critically revised the manuscript.

## Funding

This research received no specific grant from any funding agency in the public, commercial, or not‐for‐profit sectors.

## Disclosure

All authors reviewed and approved the final version of the manuscript.

## Ethics Statement

This case report was conducted in accordance with the Declaration of Helsinki. Formal institutional review board (IRB) review was waived as this constitutes a single‐patient case report, per institutional policy. Written informed consent was obtained from the patient for publication of this case report and the accompanying images.

## Consent

Please see the Ethics Statement.

## Conflicts of Interest

The authors declare no conflicts of interest.

## Supporting Information

Additional supporting information can be found online in the Supporting Information section.

## Supporting information


**Supporting Information** This case report was prepared in accordance with the CARE reporting guidelines. A completed CARE checklist is provided with this submission.

## Data Availability

Data sharing is not applicable to this article as no datasets were generated or analyzed during the current study.
