# Supplementary material for: Pulmonary Nocardia paucivorans Infection Mimicking Lung Cancer in an Immunocompetent Patient: A Case Report
Source: Case Rep Infect Dis. 2026 Jul 2;2026:4621773. doi: 10.1155/crdi/4621773 (PMC13329108; doi:10.1155/crdi/4621773)
Supplement: Supplementary file 1 — Supporting Information This case report was prepared in accordance with the CARE reporting guidelines. A completed CARE checklist is provided with this submission. [file CRDI-2026-4621773-s001.docx]

# CARE Checklist

**Supplementary File 1: Completed CARE Checklist**

Title: Pulmonary *Nocardia paucivorans* Infection Mimicking Lung Cancer in an Immunocompetent Patient: A Case Report

Authors: Mekhail M, Shiah K, Aravagiri A, Weng B, Sutjita M

Journal: Case Reports in Infectious Diseases

Manuscript ID: 4621773

| **Item** | **Checklist Item** | **Location in Manuscript** |
| --- | --- | --- |
| 1. Title | The words "case report" are included in the title | Title includes "A Case Report" verbatim. |
| 2. Keywords | 2–5 key words that identify diagnoses or interventions | Keywords section: *Nocardia paucivorans*; pulmonary nocardiosis; lung cancer mimic; MALDI-TOF MS; trimethoprim-sulfamethoxazole. |
| 3a. Abstract – Introduction | What is unique about this case and what does it add to the medical literature? | Abstract, Background section. |
| 3b. Abstract – Case Presentation | Chief complaint(s), diagnoses, interventions, and outcomes | Abstract, Case Presentation describes cough, hemoptysis, mild shortness of breath, imaging findings, diagnostic testing, tissue diagnosis, antimicrobial therapy, and follow-up outcome. |
| 3c. Abstract – Conclusion | What are the main take-away lessons from this case? | Abstract, Conclusion section. |
| 4. Introduction | Brief summary of why this case is unique, with relevant references | Section 1: Introduction: three paragraphs covering epidemiology, clinical features, extrapulmonary involvement, differential diagnosis, diagnostic approaches, therapeutic strategies, and background on *Nocardia paucivorans*. |
| 5a. Demographics | De-identified demographic and other patient-specific information | Section 2: Case Presentation: 52-year-old woman; hiking exposures in Mexico and Central California; patient de-identified. |
| 5b. Chief complaint | Main concerns and symptoms of the patient | Section 2: cough, hemoptysis, and mild shortness of breath for one month. |
| 5c. Medical/social/family history | Medical, family, and psychosocial history including relevant genetic information | Section 2: no diabetes, HIV infection, malignancy, transplantation, or chronic immunosuppressive therapy; HbA1c 5.1%; HIV negative; environmental hiking exposures documented. |
| 5d. Prior interventions | Relevant past interventions and their outcomes | Section 2, Case Presentation: no prior relevant therapeutic interventions; initial noninvasive workup, including sputum cultures, blood cultures, and acid-fast bacilli studies, was negative. |
| 6. Clinical findings | Describe physical examination findings and other significant clinical findings | Section 2, Case Presentation: physical examination significant only for bilateral scattered apical rales and crackles; the remainder was unremarkable. CT findings also described, including bilateral apical ground-glass/consolidative opacities and a spiculated pleural-based mass. |
| 7. Timeline | Important dates and times in this case | Table 2: diagnosis November 2022 through therapy completion December 2024 and ongoing surveillance. |
| 8a. Diagnostic methods | Diagnostic testing including laboratory testing, imaging, and pathology | Section 2, Case Presentation: CT imaging, sputum cultures, blood cultures, acid-fast bacilli studies, CT-guided biopsy, histopathology, Gram stain, special stains, tissue culture, MALDI-TOF MS, DNA sequencing, and broth microdilution susceptibility testing. |
| 8b. Diagnostic challenges | Diagnostic challenges | Sections 2 and 3: lung malignancy mimic, negative initial noninvasive studies, limitations of MALDI-TOF MS for rare Nocardia species, and the need for confirmatory DNA sequencing. |
| 8c. Diagnostic reasoning | Diagnostic reasoning including other diagnoses considered | Sections 2 and 3: lung malignancy, pulmonary tuberculosis, fungal infection, granulomatous infection, and pulmonary actinomycosis considered in the differential diagnosis. |
| 8d. Prognosis | Prognostic characteristics when applicable | Sections 3 and 4: prolonged therapy required; CNS involvement considered unlikely on clinical grounds; radiological follow-up planned to confirm stability and exclude occult malignancy. |
| 9a. Intervention type | Types of therapeutic intervention | Section 2, Case Presentation: pharmacological treatment with oral TMP-SMX. |
| 9b. Intervention administration | Administration of therapeutic intervention | Section 2, Case Presentation and Table 2: initial approximately 8 mg/kg/day trimethoprim component, corresponding to five double-strength tablets daily; later two double-strength tablets twice daily; total therapy from November 2022 through December 2024. |
| 9c. Intervention changes | Changes in therapeutic intervention with reasons | Section 2, Case Presentation and Table 2: attempted dose reduction to one double-strength tablet twice daily; March 15, 2024 CT showed persistent bilateral upper-lobe consolidations; dose re-escalated to two double-strength tablets twice daily. |
| 10a. Outcomes | Clinician- and patient-assessed outcomes | Section 2, Case Presentation and Table 2: hemoptysis and cough resolved; follow-up CT showed interval improvement; residual bilateral upper-lobe changes favored to represent post-inflammatory fibrosis. |
| 10b. Follow-up results | Important follow-up diagnostic and other test results | Section 2, Case Presentation and Figure 2A-B: follow-up CT demonstrated interval improvement in the dominant lesion, with residual abnormalities favored to represent post-inflammatory fibrosis. |
| 10c. Adherence/tolerability | Intervention adherence and tolerability | Section 2, Case Presentation: therapy generally well tolerated; renal function monitored periodically; mild creatinine rise attributed to trimethoprim-related inhibition of tubular creatinine secretion; no renal dose modification required; creatinine normalized. |
| 10d. Adverse events | Adverse and unanticipated events | Section 2, Case Presentation: mild creatinine rise during TMP-SMX therapy, without required treatment interruption or dose modification. |
| 11a. Strengths/limitations | Discussion of strengths and limitations of the case management approach | Section 3, Discussion: strengths include CT-guided biopsy enabling tissue diagnosis, MALDI-TOF MS with confirmatory DNA sequencing, susceptibility-guided TMP-SMX therapy, and longitudinal follow-up; limitations include absence of bronchoscopy with BAL before biopsy, unavailability of representative histopathology or microbiology images, and need for continued radiological surveillance. |
| 11b. Literature | Discussion of relevant medical literature | Section 3, Discussion; Table 3; References: Table 3 summarizes selected published Nocardia paucivorans cases; pulmonary actinomycosis addressed as a differential diagnosis; literature on pulmonary nocardiosis mimicking lung malignancy incorporated; emerging data on TMP-SMX dosing intensity and treatment duration discussed; 27 references included. |
| 11c. Rationale | Rationale for conclusions, including assessment of possible causes | Sections 2–4: tissue biopsy rationale, species-level confirmation, TMP-SMX selection based on susceptibility and outpatient feasibility, prolonged treatment based on radiographic response, and follow-up imaging to exclude occult malignancy. |
| 11d. Lessons | Primary take-away lessons of this case report | Section 4, Conclusion: maintain nocardiosis in the differential diagnosis for dominant or spiculated lung masses even in immunocompetent patients; pursue tissue diagnosis when noninvasive testing is unrevealing; confirm rare Nocardia species molecularly; perform radiological follow-up after treatment. |
| 12. Patient perspective | The patient shared their perspective or consent | Acknowledgments, Ethical Approval, and Patient Consent sections. The patient provided written informed consent for publication. |
| 13. Informed consent | The patient provided informed consent | Ethical Approval and Patient Consent section: written informed consent obtained for publication of the case report and accompanying images; IRB review waived for a single-patient case report per institutional policy. |

Checklist Reference:

Riley DS, Barber MS, Kienle GS, et al. CARE guidelines for case reports: explanation and elaboration document. J Clin Epidemiol. 2017;89:218–235. doi:10.1016/j.jclinepi.2017.04.026.

This checklist is submitted as a supplementary file in accordance with the journal requirement to follow CARE reporting guidelines for case reports.
